# Enhanced Salt Tolerance of *Torreya grandis* Genders Is Related to Nitric Oxide Level and Antioxidant Capacity

**DOI:** 10.3389/fpls.2022.906071

**Published:** 2022-05-12

**Authors:** Yang Liu, Zhuoke Jiang, Yuting Ye, Donghui Wang, Songheng Jin

**Affiliations:** ^1^Jiyang College, Zhejiang A&F University, Zhuji, China; ^2^Zhejiang Provincial Key Laboratory of Resources Protection and Innovation of Traditional Chinese Medicine, Zhejiang A&F University, Hangzhou, China

**Keywords:** antioxidant capacity, *Torreya grandis*, nitric oxide, sexual difference, salt stress

## Abstract

Nitric oxide (NO), a bioactive molecule, is often involved in the regulation of physiological and biochemical processes in stressed plants. However, the effects of NO donors on dioecious plants remain unclear. Using a pot experiment, female and male *Torreya grandis* were used to study the role of sex and NO in salt stress tolerance. In the present study, female and male *T. grandis* seedlings pretreated with an NO donor (sodium nitroprusside, SNP) were exposed to salt stress, and then leaf relative water content (RWC), photosynthetic pigments, chlorophyll fluorescence parameters, NO and glutathione levels, oxidative damage, and antioxidant enzyme activities were investigated. Female *T. grandis* plants had better tolerance to salinity, as they were characterized by significantly higher RWC, pigment content, and photochemical activities of photosystem II (PSII) and fewer negative effects associated with higher nitrate reductase (NR) activity and NO content. Pretreatment with an NO donor further increased the endogenous NO content and NR activity of both female and male *T. grandis* plants compared with salt treatment. Moreover, pretreatment with an NO donor alleviated salt-induced oxidative damage of *T. grandis*, especially in male plants, as indicated by reduced lipid peroxidation, through an enhanced antioxidant system, including proline and glutathione accumulation, and increased antioxidant enzyme activities. However, the ameliorating effect of the NO donor was not effective in the presence of the NO scavenger (Nω-nitro-L-arginine methyl ester, L-name). In conclusion, enhanced salt tolerance in *T. grandis* plants is related to nitric oxide levels and the supply of NO donors is an interesting strategy for alleviating the negative effect of salt on *T. grandis*. Our data provide new evidence to contribute to the current understanding of NO-induced salt stress tolerance.

## Introduction

It has been estimated that up to 70% of plant growth can be impacted by environmental stress, including drought, high salinity, heavy metal exposure, high or low temperatures, and light levels (Bhatnagar-Mathur et al., 2008; Mantri et al., 2012; Li et al., 2020). Salinity is a crucial factor affecting plant growth and metabolic responses throughout the world (Mostofa et al., 2015). In addition to direct ion damage, salt stress can produce secondary damage to plants through the production of reactive oxygen species (ROS) and osmotic stress (Li et al., 2020). When salt content in the soil is significantly higher than the optimal concentration for plant survival, it causes a series of stress responses in plants, including maintenance of osmotic balance and activation of the antioxidant system (Wakeel et al., 2020). In recent years, a great deal of research has been done to explore potential ways to improve saline–alkali land, including the breeding of salt-tolerant varieties, the discovery of salt-tolerant genes, and the application of hormones and growth regulators (Wang et al., 2017; Fang and Xue, 2019).

Current studies have shown that nitric oxide (NO) in plants is mainly produced through two pathways, NO synthase (NOS) and nitrate reductase (NR) pathways (Corpas and Barroso, 2017; Astier et al., 2018), and is further involved in a series of physiological and biochemical reactions, including root morphogenesis (Corpas and Barroso, 2015), seed germination (Kopyra and Gwózdz, 2003), stomatal movement (Laxalt et al., 2016), and abiotic stress regulation (Begara-Morales et al., 2014). Nitric oxide is also involved in the salt tolerance of plant growth. For example, da Silva et al. (2017) reported that salinity-induced accumulation of endogenous NO is associated with modulation of the antioxidant and redox defense systems in *Nicotiana tabacum*. Ren et al. (2020) reported that pretreatment with NO alleviates salt stress in seed germination and seedling growth of *Brassica chinensis* by enhancing biochemical parameters and regulating osmolyte accumulation. However, most of the studies focused on vegetables and herbaceous plants (Ren et al., 2020; Valderrama et al., 2021; Wani et al., 2021), and there were few studies on woody plants (Chen et al., 2021).

Dioecious plants, as an important part of the terrestrial ecosystem, account for nearly 6% of angiosperms and play an indispensable role in the cycling stability of the ecosystem (Susanne-S and Ricklefs, 1995). In the process of natural differentiation and evolution, dioecious plants evolve sex specificity and show significant differences in growth, survival, reproductive pattern, spatial distribution, and resource allocation (Todd-E and Bliss, 1989). Many researchers have devoted themselves to studying differences in the growth, survival, spatial distribution, and resource allocation of dioecious plants (Retuerto et al., 2000; Rana and Liu, 2021). On a smaller scale, some scholars have found that male and female individuals of dioecious plants show different physiological, ecological, and biochemical traits under environmental stress (Chen et al., 2018; Wang et al., 2021). For example, drought stress reduces the water content of leaves and stress resistance in female *Populus yunnanensis* (Zhang et al., 2019). In addition, the mortality rate of female plants was higher than that of male plants, indicating that the drought resistance of male *Populus yunnanensis* was stronger than that of females (Zhang et al., 2019). However, researchers have found that in some dioecious plants, females are more resistant to stress than males. For example, He et al. (2016) found that female *Ginkgo biloba* showed superior growth performance and self-protection mechanisms compared with male *Ginkgo biloba* and also had a higher photosynthetic capacity under drought stress.

*Torreya grandis* is in a genus of deciduous conifer trees and is valued for its production of nuts and timber in China, but its distribution is limited by its cultivated area and prevailing weather conditions (Hu et al., 2022; Shen et al., 2022). Many studies have investigated bioactive substance synthesis and efficacy (Hu et al., 2022; Song et al., 2022) and the interactions of functional trait × environment interactions, such as the effect of salt stress on the photosynthetic activity of *T. grandis* (Li et al., 2014). However, comparatively little research has been done to elucidate sex differences in *T. grandis* under salt stress. Our previous study indicated that *T. grandis* seedlings showed significant gender differences under drought stress, and female plants had better performance in the process of photosynthesis (Wang et al., 2021). In this study, it was hypothesized that there are sexually different responses to salt, and we also hypothesized that: (1) this difference was related to endogenous NO levels and (2) exogenous NO would improve the salt tolerance of *T. grandis* through increased endogenous NO content and antioxidant enzyme activities. To test these hypotheses, an NO donor (SNP) and NaCl were added to both male and female *T. grandis* in this study. The aims of this study were: (1) to analyze the sexually different responses of *T. grandis* to salt and (2) to evaluate the role of NO in the salt stress tolerance of *T. grandis*. The photosynthetic pigment contents, chlorophyll fluorescence parameters, leaf relative water content, levels of endogenous NO and glutathione in the leaves, and antioxidant systems were evaluated.

## Materials and Methods

### Plant Materials, Growth Conditions, and Treatments

Three-year-old *T. grandis* seedlings were obtained from the Zhuji Forestry Institute (29°43′N,120°16′E). A total of 72 seedlings with a similar mean ground diameter (10.0 mm) and seedling height (60 cm), split between males (n = 36) and females (n = 36), were used in the salt stress experiment. The trees were grown individually in plastic pots (20-cm diameter × 18-cm deep) in the greenhouse of Jiyang College of Zhejiang A&F University (Zhejiang Province, China; 29°45′N,120°15′E) in late May 2021. The greenhouse environment was controlled under a 16-h/8-h (day/night) photoperiod, with an average temperature of 30/18°С (day/night) and a relative humidity of 70 ± 5%. The plastic pots were placed in trays to prevent NaCl leaching.

Salt treatments were conducted in late June 2021. Both male and female plants were divided into four treatments: CK (control, distilled water), salt (100 mM NaCl), SNP (100 mM NaCl with 0.05 mM sodium nitroprusside [SNP]), and L-name (100 mM NaCl with 0.5 mM SNP and 0.2 mM Nω-nitro-L-arginine methyl ester [L-name]). There were three replications per treatment and three plants per replication. The NO donor (SNP) was sprayed over the surfaces of the *T. grandis* leaves three days before salt treatment (20 ml SNP per seedling). Dosage of NO was fixed according to previous studies of NO in other plants (Ren et al., 2020; Valderrama et al., 2021; Wani et al., 2021). The NO scavenger used in our study was Nω-nitro-L-arginine methyl ester (L-name) (Suda et al., 2002). To avoid osmotic shock, NaCl solution was gradually added in eight steps to achieve a concentration of 0.2%, which was related to soil weight, according to Li et al. (2014). Irrigation was conducted every three days to maintain the field capacity at 70–75%, and all indexes were measured 60 days after treatment when obvious differences were observed.

Leaves from similar positions within the mid-portion of the main stem were collected from three replicates of each treatment after analyzing the chlorophyll fluorescence parameters. After that, the leaves of each sample were cleaned and immediately used for the analysis of photosynthetic pigments, NO content and enzymatic assays.

### Determination of Photosynthetic Pigment Contents

First, finely cut leaves (collected from similar positions within the mid-portion of the main stem in each treatment) weighing 0.1 g were mixed and extracted with 8 ml 95% ethanol, according to Arnon (1949). The extraction of chlorophyll was then conducted at 4°C in the dark for 24 h and shaken about three times until the leaf samples were blanched. The absorbance of the samples was measured at 649, 665, and 470 nm using a spectrophotometer (Shimadzu UV-2550, Kyoto, Japan). The chlorophyll concentrations of *T. grandis* leaves were calculated following Arnon (1949).

### Chlorophyll Fluorescence Apparatus

The PSII photochemical activity of *T. grandis* leaves was evaluated after dark adaption for 60 min (based on the previous experiment) using a multifunctional plant efficiency analyzer (Hansatech Instruments, Pentney, UK). The fluorescent parameters, including Fv/Fm (the maximum quantum yield of the primary PSII photochemistry), ABS/RC (the absorbed light energy by the PSII antenna photon flux per active reaction center), DIo/RC (total energy dissipated by the reaction center of PSII), and TRo/RC (total energy used to restore QA by the unit reaction center of PSII), were calculated from the OJIP test curves.

### Assessment of Malondialdehyde Content and the Relative Electrolyte Leakage Rate

Lipid peroxidation was measured as the amount of malondialdehyde (MDA) determined by the thiobarbituric acid (TBA) reaction, using 0.3 g of the frozen leaf samples per replicate (n = 3) according to Deng et al. (2011). Meanwhile, 10 leaf discs (about 10 mm in diameter) from fully expanded leaves of *T. grandis* were used in each replicate (n = 3) to determine the relative electrolyte leakage conductivity (REC), according to Li et al. (2014).

### Determination of Reactive Oxygen Species

The O_2_·^−^ production rates of *T. grandis* leaves under salt and NO treatments were assessed by monitoring the nitrite formed from hydroxylamine in the presence of O_2_·^−^ as described by Wang (1990). Frozen leaf material (1 g) was used for each replicate (n = 3). Similarly, a frozen leaf (1 g) was used in each replicate (n = 3) for the analysis of H_2_O_2_ content in *T. grandis* leaves, following Patterson et al. (1984).

### Estimation of Leaf Relative Water Content

The relative water content (RWC) was measured according to the method of our previous study (Wang et al., 2021). The fresh leaves of each treatment were collected and weighed to obtain the fresh weight (FW). Leaves of *T*. *grandis* were then soaked in purified water at 25°С for 4 h to obtain the saturated fresh weight (SW). Subsequently, leaves were placed in a 70°С oven for 6 h to obtain the dry weight (DW). RWC was calculated as follows: RWC = (FW – DW)/(SW – DW) × 100%.

### Determination of Proline Content

Proline (Pro) content was determined according to Bates et al. (1973). A total of 0.3 g of *T. grandis* leaves were homogenized in sulfosalicylic acid, and then, 2 ml of acid ninhydrin and 2 ml of glacial acetic acid were added. The samples were heated at 100°C and extracted with toluene, and the free toluene was then quantified at 528 nm using *L*-proline as a standard.

### Evaluation of NO and Nitrate Reductase

Fresh leaves (0.3 g) were used in each replicate (n = 3) for the analysis of NO content, according to Cantrel et al. (2011). NO content was calculated using a standard curve of NaNO_2_ (0–4 μg mL^−1^) and expressed as μmol g^−1^ FW. The activity of nitrate reductase (NR; E.C. 1.6.6.1) was determined according to Jaworski (1971). Similarly, a fresh leaf (0.3 g) was used for each replicate (n = 3). The activity of NR was expressed as nmol NO_2_^−1^ min^−1^ mg^−1^ protein.

### Protein Extraction, GSH Content, and Antioxidant Enzymes

Antioxidant enzyme extracts from each treatment were obtained from 0.3 g frozen leaves, according to Yu et al. (2017). The leaves were homogenized at 4°C in 10 ml of 50 mM phosphate buffer solution (pH 7.8) containing 1% polyethylenepyrrole. The homogenate was then centrifuged at 10,000 × g at 4°C for 15 min. The supernatant was collected and used to measure GSH content and enzyme activity. Meanwhile, soluble proteins were determined according to Bradford (1976).

GSH content and glutathione reductase (GR) activity were assayed using kits from the Nanjing Jiancheng Bioengineering Institute (Nanjing, China), following the manufacturer’s specifications. GR activity was expressed as nmol min^−1^ mg^−1^ protein. The GSH content was expressed as mg g^−1^ FW. SOD activity was analyzed following Beauchamp and Fridovich (1971) and was expressed as U g^−1^ protein. POD activity was estimated according to Ruan et al. (2002) and was expressed as U mg^−1^ protein. CAT activity was measured following Watanabe et al. (2003) and was expressed as μmol min^−1^ mg^−1^ protein. APX activity was measured using the method described by Nakano and Asada (1981) and was expressed as nmol min^−1^ mg^−1^ protein.

### Statistical Analyses

Two-way analysis of variance (ANOVA), with sex and salt as the main factors and a sex × salt interaction term, was performed using SPSS 19.0 (SPSS Inc., Chicago, IL, USA) to evaluate the effects of sex and salt treatments and was followed by Duncan’s test (*p* < 0.05). The data are presented as the mean ± standard deviation (SD).

## Results

### Effects of Sex and Salt on Chlorophyll Contents and Chlorophyll Fluorescence

**Figure 1:**
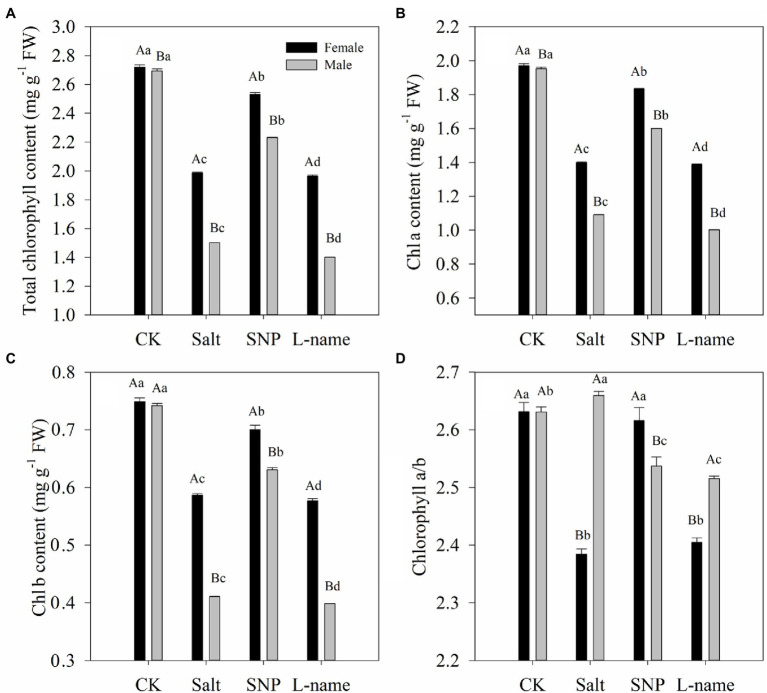
Photosynthetic pigment contents [total chlorophyll content **(A)**, Chl a content **(B)**, Chl b content **(C)**, and ratio of Chl a to Chl b **(D)**] measured in *T. grandis* in response to different treatments. CK (control, distilled water), salt (100 mM NaCl), SNP (100 mM NaCl with 0.05 mM SNP), and L-name (100 mM NaCl with 0.5 mM SNP and L-name). Different uppercase letters indicate a significant difference between different genders at *p* < 0.05, and different lowercase letters indicate significant difference under salt treatment at *p* < 0.05, according to Duncan’s test.

**Figure 2:**
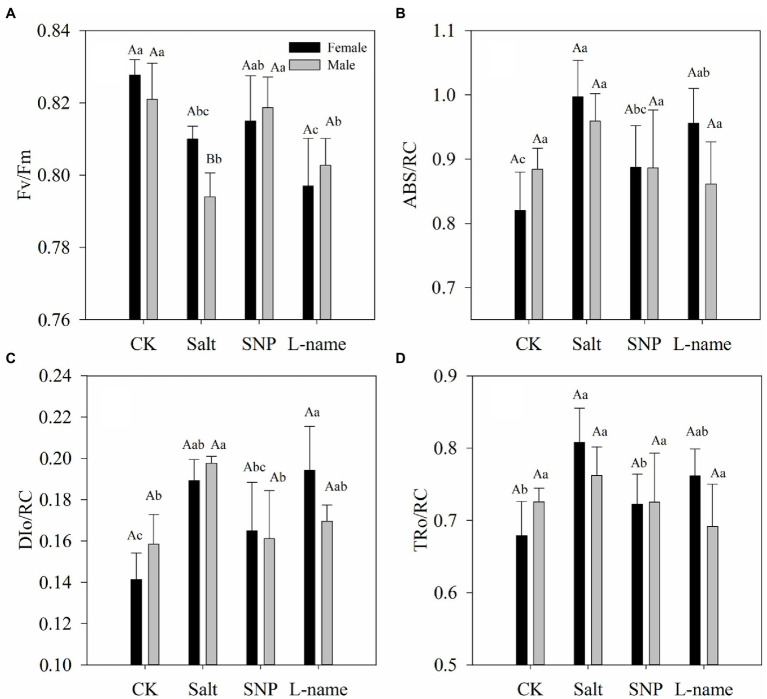
Chlorophyll fluorescence parameters (Fv/Fm: **A**; ABS/RC: **B**, DIo/RC: **C**, TRo/RC: **D**) measured in *T. grandis* in response to different treatments. CK (control, distilled water), salt (100 mM NaCl), SNP (100 mM NaCl with 0.05 mM SNP), and L-name (100 mM NaCl with 0.5 mM SNP and L-name). Different uppercase letters indicate a significant difference between different genders at *p* < 0.05, and different lowercase letters indicate significant difference under salt treatment at *p* < 0.05, according to Duncan’s test.

The chlorophyll contents and chlorophyll fluorescence parameters of *T. grandis* were found to be significantly different under salt treatments (Table 1). Moreover, a significant interaction of sex and salt was observed in the total chlorophyll content, Chl a content, Chl b content, and ratio of Chl a to Chl b of *T*. *grandis* (Table 1). Compared with the control, salt treatment significantly inhibited the growth of both T. *grandis* genders, which was evident from lower pigment contents and Fv/Fm values but higher ABS/RC, DIo/RC, and TRo/RC values (Figures 1, 2). Meanwhile, compared with male plants, female plants grown under salt were characterized by significantly higher Fv/Fm and pigment contents and a smaller Chl a/b ratio (Figures 1, 2). In comparison with salt-treated seedlings, SNP treatment caused an increase in pigment contents and PSII activity in both female and male *T*. *grandis* (Figures 1, 2). However, inclusion of NO scavenger (L-name) decreased this effect and again caused a reduction in Fv/Fm and pigment contents and also increased the values of ABS/RC, DIo/RC, and TRo/RC (Figures 1, 2).

**Table 1:** Summary of significance levels (two-way ANOVA) for the effects of gender, salt, and their interaction on the contents of photosynthetic pigments and fluorescence parameters.

Source		Total chlorophyll content (mg g^−1^ FW)	Chl a content (mg g^−1^ FW)	Chl b content (mg g^−1^ FW)	Chlorophyll a/b	Fv/Fm	ABS/RC	DIo/RC	TRo/RC
Gender (A)	MS	8,444.21	9,485.13	3,911.02	217.46	0.84	0.47	0.01	0.75
	P	0.0001	0.0001	0.0001	0.0001	0.3730	0.4958	0.9130	0.4002
Salt (B)	MS	17,773.85	23,560.85	5,420.36	199.65	10.53	4.63	8.62	3.43
	P	0.0001	0.0001	0.0001	0.0001	0.0005	0.0163	0.0012	0.0426
Interaction (A × B)	MS	1,016.62	1,057.87	596.68	218.37	1.90	1.81	1.87	1.86
	P	0.0001	0.0001	0.0001	0.0001	0.1699	0.1859	0.1743	0.1774

### Effects of Sex and Salt on the Level of Lipid Peroxidation

**Figure 3:**
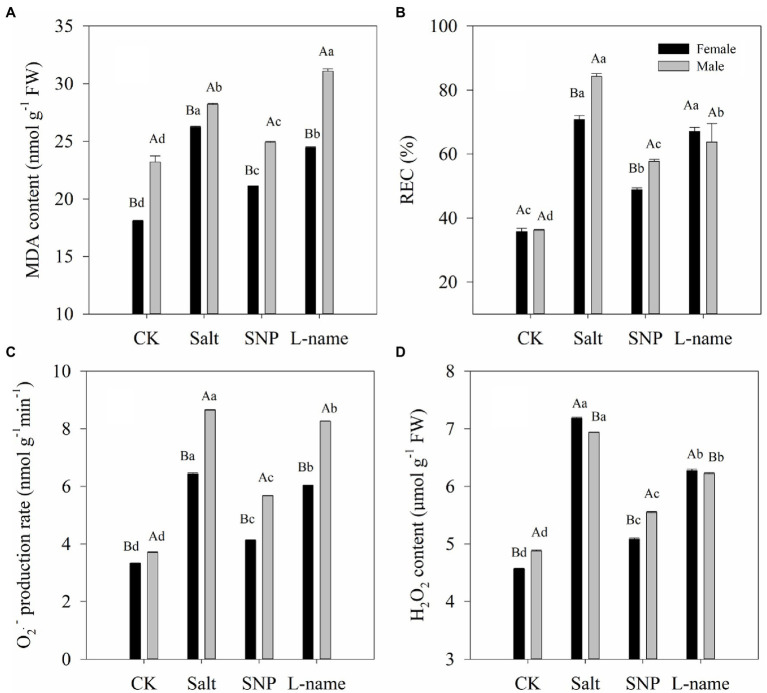
Lipid peroxidation [MDA content **(A)**, REC **(B)**, O2·- production rate **(C)**, H2O2 content **(D)**] of *T. grandis* leaves in response to different treatments. CK (control, distilled water), salt (100 mM NaCl), SNP (100 mM NaCl with 0.05 mM SNP), and L-name (100 mM NaCl with 0.5 mM SNP and L-name). Different uppercase letters indicate a significant difference between different genders at *p* < 0.05, and different lowercase letters indicate significant difference under salt treatment at *p* < 0.05, according to Duncan’s test.

The effects of salt and NO donors on membrane damage of cells were analyzed by determining the MDA content and the relative electrolyte leakage rate, while oxidative stress was investigated in terms of the O_2_·^−^ production rate and H_2_O_2_ content. Values of the MDA content, the relative electrolyte leakage rate, the O_2_·^−^ production rate, and H_2_O_2_ content of *T*. *grandis* were all significantly affected by sex, salt, and their interaction (Table 2; *p* < 0.05). As shown in Figure 3, compared with the control, salt treatment significantly increased the MDA content, relative electrolyte leakage rate, O_2_·^−^ production rate, and H_2_O_2_ content of *T*. *grandis* (*p* < 0.05). Meanwhile, compared with female plants, male plants grown under salt stress were characterized by significantly higher MDA content, relative electrolyte leakage rate, and O_2_·^−^ production rate but reduced H_2_O_2_ content (Figure 3; *p* < 0.05). However, pretreatment with SNP resulted in significant decreases in these indexes compared with the salt-treated female and male plants (*p* < 0.05), but this was decreased by the NO scavenger under the L-name treatment.

**Table 2:** Summary of significance levels (two-way ANOVA) for the effects of gender, salt, and their interaction on RWC, proline content, the level of lipid peroxidation, NO content and NR activity, GSH content and GR activity, and antioxidant enzyme activities.

Source		RWC (%)	Proline content (μg g^−1^ FW)	MDA content (nmol g^−1^ FW)	REC (%)	O_2_·^−^ production rate (nmol g^−1^ min^−1^)	H_2_O_2_ content (μmol g^−1^ FW)	NR activity (nmol min^−1^ mg^−1^ protein)
Gender (A)	MS	4.51	1719.60	2049.20	28.90	36716.53	285.55	18.31
	P	0.0497	0.0001	0.0001	0.0001	0.0001	0.0001	0.0006
Salt (B)	MS	37.82	58628.69	921.47	379.65	52280.93	21405.36	281.25
	P	0.0001	0.0001	0.0001	0.0001	0.0001	0.0001	0.0001
Interaction (A × B)	MS P	11.67 0.0003	121.59 0.0001	831.53 0.0001	17.75 0.0001	2766.38 0.0001	530.26 0.0001	31.92 0.0001
Source		NO content (μmol g^−1^ FW)	GR activity (nmol min^−1^ mg^−1^ protein)	GSH content (mg g^−1^ FW)	SOD activity (U g^−1^ protein)	POD activity (U mg^−1^ protein)	CAT activity (μmol min^−1^ mg^−1^ protein)	APX activity (nmol min^−1^ mg^−1^ protein)
Gender (A)	MS	35.62	112.59	1423.36	80.40	372.89	199.27	195.98
	P	0.0001	0.0001	0.0001	0.0001	0.0001	0.0001	0.0001
Salt (B)	MS	2476.18	111.60	23448.59	353.36	281.31	644.22	141.27
	P	0.0001	0.0001	0.0001	0.0001	0.0001	0.0001	0.0001
Interaction (A × B)	MS P	163.24 0.0001	19.83 0.0001	425.80 0.0001	93.39 0.0001	28.85 0.0001	81.52 0.0001	25.87 0.0001

### Effects of Sex and Salt on RWC and Proline Content

**Figure 4:**
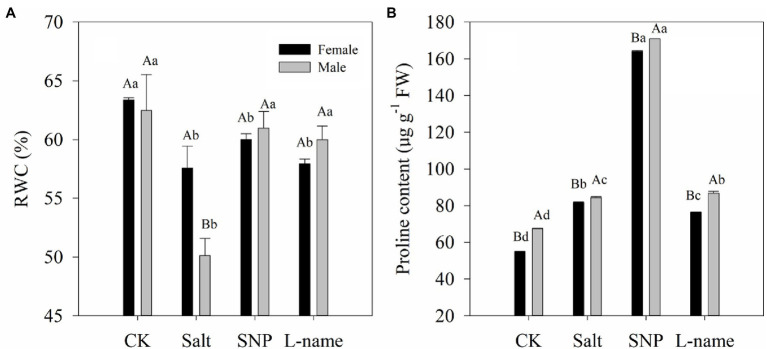
Leaf relative water (RWC) content **(A)** and proline content **(B)** of *T. grandis* in response to different treatments. CK (control, distilled water), salt (100 mM NaCl), SNP (100 mM NaCl with 0.05 mM SNP), and L-name (100 mM NaCl with 0.5 mM SNP and L-name). Different uppercase letters indicate a significant difference between different genders at *p* < 0.05, and different lowercase letters indicate significant difference under salt treatment at *p* < 0.05, according to Duncan’s test.

The physiological water status and proline content of *T*. *grandis* plants are shown in Figure 3. Both the RWC and proline content of *T*. *grandis* were significantly affected by sex, salt, and their interaction (Table 2; *p* < 0.05). Compared with the control, the RWC in salt-treated *T. grandis* leaves decreased by 9.1% (*p* < 0.05) in female plants and 19.8% (*p* < 0.05) in male plants. The NO treatment reduced the effect of salt stress on the RWC decrease in the presence of 0.2% NaCl, and it was significant in the male plants (Figure 4A). In contrast, the inclusion of the NO scavenger (L-name) decreased the effect of SNP on the water status and caused a reduction in RWC; however, these changes were not significant (*p* > 0.05; Figure 4A). As shown in Figure 4B, the proline content of both female and male *T*. *grandis* significantly increased under both salt and SNP treatments compared with that of CK. However, this was significantly decreased by the NO scavenger under the L-name treatment.

### Effects of Sex and Salt on NO Content and NR Activity

**Figure 5:**
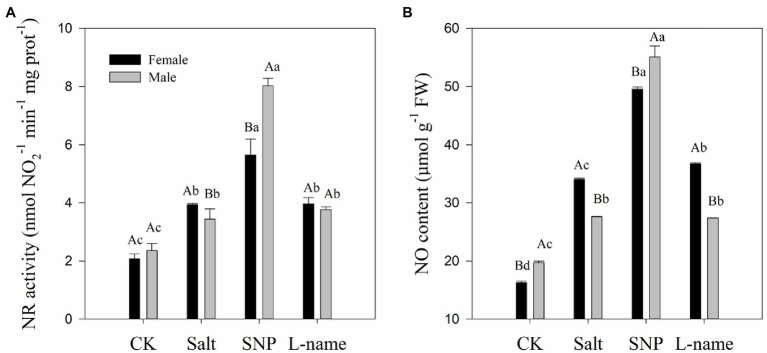
Nitrate reductase (NR) activity **(A)** and NO content **(B)** of *T. grandis* in response to different treatments. CK (control, distilled water), salt (100 mM NaCl), SNP (100 mM NaCl with 0.05 mM SNP), and L-name (100 mM NaCl with 0.5 mM SNP and L-name). Different uppercase letters indicate a significant difference between different genders at *p* < 0.05, and different lowercase letters indicate significant difference under salt treatment at *p* < 0.05, according to Duncan’s test.

Two-way ANOVA showed that both the NR activity and NO content of *T. grandis* were significantly affected by sex, salt, and their interaction (Table 2; *p* < 0.05). Compared with male plants, female plants grown under salt were characterized by significantly higher NR activity and endogenous NO content (Figure 5; *p* < 0.05). As shown in Figure 5, compared with the control, salt treatment significantly increased the NR activity and endogenous NO content of *T*. *grandis* by 89.7 and 109.2%, respectively, in female plants and by 45.8 and 40.3%, respectively, in male plants. However, pretreatment with SNP before salt treatment resulted in a further increase in the NR activity (43.2 and 133.6% in female and male plants, respectively) and NO content (45.6 and 99.7% in female and male plants, respectively) in *T. grandis* leaves compared with the salt-treated plants (*p* < 0.05; Figure 5). However, the inclusion of the NO scavenger decreased the effect of SNP on NO synthesis, as it was evident that the L-name treatment reduced NR activity by 29.7 and 53.1% and NO content by 25.9 and 50.3% in female and male plants, respectively, compared to salt-stressed plants treated with SNP (*p* < 0.05; Figure 5).

### Effects of Sex and Salt on Endogenous GSH Content and GR Activity

**Figure 6:**
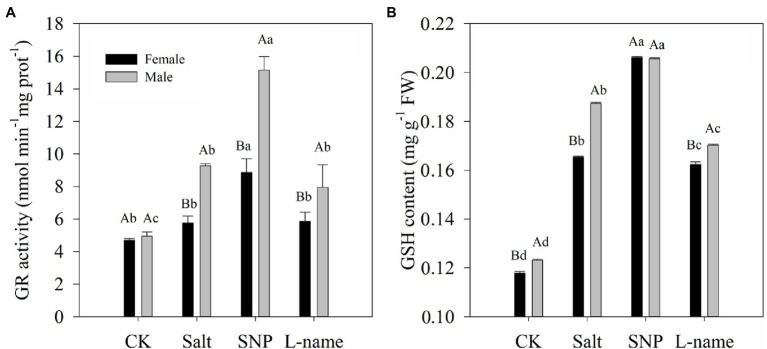
Glutathione reductase (GR) activity **(A)** and GSH content **(B)** of *T. grandis* in response to different treatments. CK (control, distilled water), salt (100 mM NaCl), SNP (100 mM NaCl with 0.05 mM SNP), and L-name (100 mM NaCl with 0.5 mM SNP and L-name). Different uppercase letters indicate a significant difference between different genders at *p* < 0.05, and different lowercase letters indicate significant difference under salt treatment at *p* < 0.05, according to Duncan’s test.

Figure 6 illustrates the endogenous GSH content and the activity of the key enzyme involved in GSH biosynthesis under different treatments. Two-way ANOVA showed that both GR activity and GSH content of *T. grandis* were significantly affected by sex, salt, and their interaction (Table 2; *p* < 0.05). Compared with female plants, male plants grown under salt stress were characterized by significantly higher GR activity and GSH content (Figure 6; *p* < 0.05). As shown, compared with the control, salt treatment increased the GR activity and GSH content of *T*. *grandis* by 22.5% (*p* > 0.05) and 40.3% (*p* < 0.05), respectively, in female plants and by 87.6% (*p* < 0.05) and 52.1% (*p* < 0.05), respectively, in male plants. However, pretreatment with an NO donor before salt treatment resulted in a further increase in GR activity (47.1 and 24.6% in females and males, respectively) and GSH content (63.3 and 97.7% in females and males, respectively) in *T*. *grandis* leaves relative to the salt-stressed plants (*p* < 0.05; Figure 6). However, the inclusion of L-name decreased the effect of NO on GSH synthesis.

### Effects of Sex and Salt on Antioxidant Enzyme Activity

**Figure 7:**
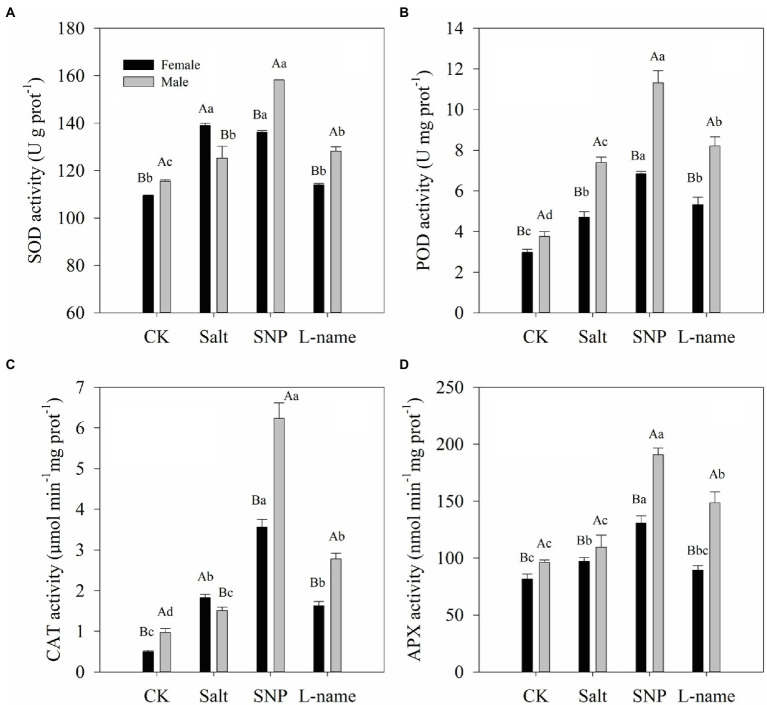
Antioxidant enzyme activities of *T. grandis* in response to different treatments. CK (control, distilled water), salt (100 mM NaCl), SNP (100 mM NaCl with 0.05 mM SNP), and L-name (100 mM NaCl with 0.5 mM SNP and L-name). Different uppercase letters indicate a significant difference between different genders at *p* < 0.05, and different lowercase letters indicate significant difference under salt treatment at *p* < 0.05, according to Duncan’s test.

Two-way ANOVA showed that the antioxidant enzyme activities of *T. grandis* were significantly affected by sex, salt, and their interaction (Table 2; *p* < 0.05). As shown in Figures 7A–D, the activities of the studied antioxidant enzymes significantly increased under salt treatment compared with the control plants. Compared with male plants, female plants grown under salt stress were characterized by significantly higher SOD and CAT activities but lower POD and APX activities (Figure 7; *p* < 0.05). Pretreatment with an NO donor before salt treatment further increased SOD (by 26.3% in males), POD (by 45.3 and 53.0% in females and males, respectively), CAT (by 94.8 and 312.8% in females and males, respectively), and APX (by 34.8 and 74.1% in females and males, respectively) activities compared with the salt treatment. However, the inclusion of L-name decreased the effect of NO on antioxidant enzyme activities (Figure 7). Thus, these results suggest that the modulation of antioxidant enzyme activities by an exogenous NO donor might alleviate the ROS-triggered oxidative damage caused by salt stress.

## Discussion

In recent years, studies have shown that NO is involved in plant morphological development, root growth, photosynthesis, and the regulation of antioxidant enzyme systems, especially when plants are subjected to environmental stress (Valderrama et al., 2021; Wani et al., 2021), but few studies have focused on the effects of NO donors on dioecious plants. This study confirmed our hypothesis that NO is involved in the salt-tolerant growth of both female and male *T*. *grandis*. Salt treatment inhibited the growth of female and male *T. grandis* seedlings, which was indicated by a lower photosynthetic pigment content and decreased Fv/Fm but significantly higher ABS/RC, DIo/RC, and TRo/RC (Figures 1, 2). Additionally, compared with male plants, female plants showed better tolerance to salinity, as they were characterized by significantly higher Fv/Fm and pigment contents and fewer negative effects (Figures 1–3). Our results are similar to those of previous drought tests on *T*. *grandis* (Wang et al., 2021), suggesting that female *T*. *grandis* are more tolerant of stress. However, a study by Li et al. (2013) on *Populus yunnanensis* reported that females were more sensitive and suffered from greater negative effects than males under salt stress. Therefore, these studies suggest that sex-linked differences in the growth of dioecious plants are not consistent among species, which can be interpreted as species-specific adaptation. Pretreatment with an NO donor (SNP) caused an increase in pigment contents and PSII activity of both female and male *T*. *grandis*; however, inclusion of the NO scavenger (L-name) decreased this effect (Figures 1, 2). Thus, these results suggest that pretreatment with NO donors is a simple and effective measure to reduce the effect of salt on the growth of *T*. *grandis*. These findings are consistent with previous reports on saffron (Babaei et al., 2021), *Arabidopisis thaliana* (Liu et al., 2015), and *Glycine max* (Vaishnav et al., 2016).

Chlorophyll is an important factor in photosynthesis in plants. In this study, salt and NO treatments significantly affected chlorophyll pigments and their ratios (Figure 1). Decreases in total chlorophyll content of both female and male *T. grandis* were observed under salt treatment, suggesting that salt stress caused damage to photosynthetic pigments. Similar results were also reported in *Mentha spicata* and *Oryza sativa* (Amri et al., 2011; Ounoki et al., 2021). However, compared with salt treatment, NO treatment in both female and male *T*. *grandis* resulted insignificantly higher leaf chlorophyll contents, which was inconsistent with that reported in mustard and cotton (Khan et al., 2012; Liu et al., 2013). Changes in the Chl a/b ratio often indicate a change in the ratio between the light-harvesting complexes (LHC) and the reaction center complexes of the photosystem (Venema et al., 2000). The significantly increased Chl a/b ratio in both female and male *T*. *grandis* under NO treatment showed that NO might participate in inducing these plants to change the ratio of chlorophyll pigments to maintain a good photoelectron transfer system. This was confirmed by the supply of the NO scavenger (L-name), which reversed the effect of SNP on pigment accumulation and caused a reduction in total chlorophyll, Chl a, and Chl b contents (Figure 2).

Chlorophyll fluorescence parameters are often used to reflect the photosystem changes of plants in the face of environmental stress, and the measurement is convenient, accurate, and sensitive (Pashkovskiy et al., 2018). Generally, plants grown under stress have lower Fv/Fm than non-stressed plants (Baker, 2008), which is consistent with the performance of *T*. *grandis* (Figure 2). The significantly higher values of Fv/Fm under treatment with an NO donor compared with those under salt treatment indicated that pretreatment with an NO donor can effectively maintain photochemical efficiency in *T*. *grandis* seedlings. The increase in ABS/RC in both female and male *T*. *grandis* under salt treatment can be explained by a decrease in the number of active reactive centers (RC) of PSII, which might serve as a defense mechanism to reduce the burden of its systems when salt stress occurs (Strasser et al., 2010). Another effective protective mechanism for plants under salt stress is the dissipation of the absorbed light energy into heat (Baena-Gonzalez and Aro, 2002), which was confirmed by the significantly higher values of non-photochemical quenching (NPQ) per reaction center of PSII (DIo/RC) in *T*. *grandis* seedlings under salt stress (Figure 2C). Furthermore, TRo/RC was significantly higher under salt, which indicated that *T. grandis* seedlings under salt stress had improved the efficiency of the remaining active reaction centers. However, compared with salt-treated seedlings, NO treatment caused an increase in PSII activity in both female and male *T*. *grandis* (Figure 2), which is indicated by higher Fv/Fm and lower ABS/RC, DIo/RC, and TRo/RC. However, the inclusion of an NO scavenger (L-name) decreased this effect, caused a reduction in Fv/Fm, and increased the values of ABS/RC, DIo/RC, and TRo/RC (Figure 2). Together, these results confirm that the supply of an NO donor can maintain the photosynthetic capacity of *T*. *grandis* plants under salt stress.

When plants are stressed, the photosynthetic electron transport system produces a large number of reactive oxygen species (ROS), including O_2_·^−^ and H_2_O_2_ (You and Chan, 2015). This is consistent with the significantly increased values of O_2_·^−^ and H_2_O_2_ in the leaves of *T*. *grandis* under salt stress (Figure 3). Furthermore, the accumulation of ROS could lead to a break in the osmotic balance of plant cell membranes and eventually to membrane lipid peroxidation (Golldack et al., 2014). The increase in MDA content often indicates the occurrence of membrane lipid peroxidation, while the change in membrane permeability leads to an increase in relative conductivity (Li et al., 2014). Compared with female plants, male *T*. *grandis* seedlings grown under salt suffered from more negative effects, as they were characterized by a significantly higher MDA content, relative electrolyte leakage rate, and O_2_·^−^ production rate but decreased H_2_O_2_ content (Figure 3; *p* < 0.05). These results indicated that female *T*. *grandis* were more tolerant to environmental stress than male *T*. *grandis*, which is consistent with previous results using a drought test (Wang et al., 2021). Our results also showed that the addition of SNP could effectively maintain the balance of intracellular and intracellular permeable substances and the stability of the cell membrane in both male and female *T*. *grandis* (Figure 3). This was also confirmed by the application of the NO scavenger (L-name), which again increased these parameters. These results were relatively consistent with the results in *Brassica oleracea* (Akram et al., 2020), broccoli (Ahmad et al., 2016), and cotton (Dong et al., 2014).

RWC commonly exhibits a decreasing trend under salt treatment (Li et al., 2014). In this study, we found that values of RWC declined under NaCl treatment, and sexual differences were significant, indicating that the leaf water situation of male and female *T*. *grandis* are quite different under salt stress. Compared with males, female *T*. *grandis seedlings* maintained significantly higher RWC under salt stress (Figure 4A), indicating that female *T*. *grandis* seedlings are more tolerant to salt stress than male plants. When plants suffer from salt stress, the accumulation of osmotic substances and proline content in leaf cells is often used as an effective means of alleviating stress. Both male and female *T*. *grandis* seedlings showed a higher accumulation of proline under salt stress, while SNP treatment further increased proline content and RWC (Figure 4). In contrast, the inclusion of the NO scavenger (L-name) decreased the effect of SNP on proline accumulation and caused a reduction in RWC, which confirmed that NO might be involved in maintaining the water content in *T*. *grandis*. A similar result was reported by Chen et al. (2021), who found that H_2_S-induced NO alleviated the salt stress of Cyclocarya paliurus by improving proline accumulation.

The synthesis of NO increases when plants are subjected to stress, which has been reflected in this work and in previous research (Valderrama et al., 2021; Wani et al., 2021). Meanwhile, the results of this study showed that the activity of NR and NO content increased simultaneously, indicating that NR is an important enzyme in the synthesis of NO in *T. grandis* (Corpas and Barroso, 2017). NR-mediated NO production has been reported to be involved in plant physiological responses under stress (Mur et al., 2013). Studies on wheat seedlings have also indicated that the application of NO donors could significantly increase both NR activity and NO content (Khan et al., 2017). Compared with male *T*. *grandis*, female *T*. *grandis* induced significantly higher NR activity and NO content under salt stress, which to some extent explained the physiological and biochemical differences between male and female *T*. *grandis* under environmental stress. However, compared with females, male *T*. *grandis* treated with exogenous SNP synthesized more NO (Figure 5B), which may help male *T*. *grandis* alleviate the damage caused by salt stress, as they have a lower tolerance. The inclusion of the NO scavenger confirmed the effect of the external SNP supply on endogenous NO synthesis, which was consistent with the results of cotton (Dong et al., 2014) and *Zea mays* (Kharbech et al., 2020).

One of the mechanisms through which NO participates in plant stress resistance is the activation of the antioxidant system, including the non-enzymatic and enzymatic antioxidant systems (da-Silva, C. J., Modolo, L. V., 2017; Wani et al., 2021). A large number of studies have shown that the content of non-enzymatic substances, such as phenolics and soluble proteins, in salt-treated plants can be significantly increased with the participation of NO donors (Ahmad et al., 2016; da-Silva, C. J., Modolo, L. V., 2017; Chen et al., 2021). The results of this study showed that the GSH content and GR activity of *T*. *grandis* seedlings under salt stress were significantly increased after treatment with an NO donor (Figure 6). This result is consistent with previous studies on wheat (Hasanuzzaman et al., 2011), cotton (Liu et al., 2013), and chickpea (Ahmad et al., 2016). Our previous study also showed that NO induced by hydrogen sulfide was involved in the regulation of the salt tolerance of *T*. *grandis*, which was related to the massive synthesis of different phenolic antioxidants (Chen et al., 2021). In general, the accumulation of non-enzymatic substances is beneficial for plant cells to cope with osmotic damage caused by stress.

The production of antioxidant enzymes, such as SOD, POD, CAT, and APX, in plants often keeps ROS in a stable state, and these antioxidant systems are activated when ROS are greatly increased under stress (Wakeel et al., 2020). O_2_·^−^ is dissimulated to H_2_O_2_ by SOD, which is then eliminated by CAT and APX, producing H_2_O and O_2_ (Lázaro et al., 2013). Female *T*. *grandis* under salt stress showed significantly higher SOD and CAT enzyme activities, while male T. *grandis* showed significantly higher POD and APX enzyme activities (Figure 7). This also explains the different responses of male and female *T*. *grandis* to salt stress. Compared with female *T*. *grandis*, salt-stressed male *T*. *grandis* had significantly higher SOD, POD, CAT, and APX enzyme activities after SNP treatment. This suggests that NO might be more effective in alleviating the salt stress of male *T*. *grandis* by improving the activities of antioxidant enzymes. These results also indicated that the increase in the activity of these antioxidant enzymes mediated by SNP might be due to post-translational modifications (PTM), such as persulfidation or S-nitrosation (Singh et al., 2017; Alamri et al., 2020).

Overall, our study showed that female *T*. *grandis* showed better tolerance to salinity, as they were characterized by significantly higher RWC, pigment contents, and Fv/Fm and fewer negative effects. This is associated with higher nitrate reductase (NR) activity and NO content. Pretreatment with an NO donor alleviated salt-induced oxidative damage of *T*. *grandis*, especially male *T*. *grandis*, as indicated by lowered lipid peroxidation through an enhanced antioxidant system, including proline and glutathione accumulation, and increased antioxidant enzyme activities. However, the ameliorating effect of the NO donor was not effective in the presence of an NO scavenger. The present study provides new evidence to contribute to the current understanding of NO-induced salt stress tolerance and the role of NO signaling in dioecious plants.

## Data Availability Statement

The raw data supporting the conclusions of this article will be made available by the authors, without undue reservation.

## Author Contributions

YL is responsible for the whole process of experimenting and writing the paper. SJ provide experimental guidance and amend the manuscript. JK, YT, and DW help to do the experiment. All authors contributed to the article and approved the submitted version.

## Funding

This work was supported by the National Key Research and Development Project (2019YFE0118900), the National Natural Science Foundation of China (No. 32001305), Jiangsu Province Natural Science Foundation (No. BK20200771), “Pioneer” and “Leading Goose” R&D Program in Universities of Zhejiang (2022), and Jiyang College of Zhejiang A & F University under Grant (No. RQ2020B14; JYKC2117).

## Conflict of Interest

The authors declare that the research was conducted in the absence of any commercial or financial relationships that could be construed as a potential conflict of interest.

## Publisher’s Note

All claims expressed in this article are solely those of the authors and do not necessarily represent those of their affiliated organizations, or those of the publisher, the editors and the reviewers. Any product that may be evaluated in this article, or claim that may be made by its manufacturer, is not guaranteed or endorsed by the publisher.
